# Editorial: Mining, Designing, Mechanisms and Applications of Extremophilic Enzymes

**DOI:** 10.3389/fmicb.2021.709377

**Published:** 2021-10-25

**Authors:** Massimiliano Fenice, Sunil Kumar Khare, Susanna Gorrasi

**Affiliations:** ^1^Dipartimento di Scienze Biologiche ed Ecologiche, University of Tuscia, Viterbo, Italy; ^2^Department of Chemistry, Indian Institute of Technology Delhi, New Delhi, India

**Keywords:** extremophiles, extremozymes, enzyme mining, enzyme purification, enzyme characterization, enzyme structure, biotechnology applications, protein engineering

Enzymes play very important roles in industrial and environmental biotechnology. Their value has increased in recent decades mainly in food and detergent industries and is still growing, particularly in bioremediation (white biotechnology), and in medical, pulp/paper, textile, energy, and biosensor applications (Fernández-Lucas et al., 2017). Recent assessments have evaluated the global enzyme market at more than $7,000 million, and the business is estimated to rapidly exceed $10,000 million (Fernández-Lucas et al., 2017; Pasqualetti et al., 2019). Enzyme technologies provide economically viable and eco-friendly alternatives. The number of commercial enzymes continues to increase as we exploit, by traditional or molecular screening/selection methods, the astonishing microbial diversity to obtain new bio-catalysts and expand their range of application (Petruccioli et al., 1993; Xiao et al., 2015; Sysoev et al., 2021). Most extremophilic enzymes (extremozymes) come from microorganisms. Extremozymes remain active and stable in extreme conditions, such as high pH values, high salinity and hydrostatic pressure, and temperature extremes. They often have unique properties empowering them to satisfy the needs of many harsh industrial processes. From a commercial viewpoint, enzymes with high activity and adaptation to diverse extreme conditions show growing demand, since current applications of many existing enzymes are still constrained by activity, stability, and/or economic issues. One solution to this problem is searching for new enzymes from natural sources that show high activity/stability, uncommon specificity, and poly-extremophilic features (thermo/psychrophilic, acid/alkalophilic, and halophilic properties). However, not all extremozymes are isolated from extreme environments. Another winning strategy, which is expected to improve enzyme properties for biotechnological application, is protein engineering (Xiao et al., 2015; Liu et al., 2019). The objective of this Research Topic was to inspect new details in this growing research field, and to obtain a deeper understanding of the area, allowing us to outline its state-of-the-art: by showing enzymes that have high activity and stability in extreme or poly-extreme conditions; understanding their relative adaptation mechanisms; reporting successful mining, designing, characterization, and production of novel extremophilic enzymes also by bioinformatic tools. We cover 15 manuscripts that explore different aspects of this Research Topic.

To improve the thermostability of lipases from *Rhizopus chinensis*, Jiang et al. identified mutagenesis sites associated with enhanced flexibility based upon B-factor analysis and multiple sequence alignment. Two isoforms exhibited enhanced thermostability and improved residual activity. Novel highly thermostable mutant lipases for industrial applications can be predicted by B-factor analysis and constructed via site-directed mutagenesis.

Yang et al. purified and characterized a thermostable laccase from *Trametes trogii* strain S0301. The enzyme matched the sequences of lcc3 in *T. trogii* BAFC463. It showed efficient dye decolorization at 60°C. This is the first report of a thermo-activated laccase from a thermophilic *Trametes trogii* strain having better properties among fungal laccases, with prospective applications in biotechnology.

Zhou et al. are reporting the heterologous expression of an invertase gene (GspInv) of *Gongronella* sp. w5 in *Komagataella pastoris*. The enzyme was characterized and shown to be an ideal candidate for high fructose syrup production with a high conversion efficiency (95%). When immobilized on cellulose, the protein retained the properties of invertase GspInv, suggesting that it could be a promising invertase for high fructose syrup preparation.

Perfumo et al. characterized enzymatic activities of a psychrophilic Antarctic strain of *Psychrobacter* sp., producing lipases and proteases. The sequence of an extracellular serine-protease was identified across *Psychrobacter* sp. genomes, and expressed in *Escherichia coli*. The purified enzyme was a cold-active alkaline protease that was found to be stable in presence of common inhibitors, compatible with detergents, and therefore suitable for new generation cold washing products.

A study by Yang et al. identified and characterized enzymes conferring salt tolerance to gut microbes. By screening the fecal metagenomic library, 10 out of the 48 salt-tolerant clones detected exhibited stronger tolerance and stability at different NaCl concentrations. High-throughput sequencing analysis showed that 91 genes encoded proteins involved in salt tolerance. Two trehalose-6-phosphate hydrolase genes were expressed in *Escherichia coli* and characterized. They were salt-tolerant and their activity increased with increasing NaCl concentration. The results indicate the existence of numerous salt-tolerant genes in gastrointestinal microbes, providing new insights into their salt-tolerance mechanisms.

Li et al. characterize the first cocrystal structure of sucrose phosphate synthase, a rate-limiting enzyme in sucrose synthesis, from *Thermosynechococcus elongatus* with UDP and sucrose-6-phosphate was characterized. It was observed that two sucrose phosphate synthase mutants lost all catalytic activity. Moreover, temperature gradient analysis shows that the enzyme exhibits the highest activity at 70°C, suggesting that it has potential uses in the industrial production of sucrose-6-phosphate.

Bhatt and Singh expressed an alkaline protease gene of *Bacillus lehensis* from a saline desert habitat in *Escherichia coli*. The recombinant protease (APrBL) belongs to the subtilase S8 protease family. After characterization, the APrBL protease turned out to be distinct from well-known commercial proteases and highly effective as a detergent additive and in whey protein hydrolysis.

The review by Zhang et al. highlights new aspects of DNA in hyperthermophilic Archaea that thrive in high-temperature environments. Although their genome is facing severe stability challenges due to the increased DNA damages by high temperature, hyperthermophilic Archaea display mutation frequencies similar to mesophilic microorganisms, indicating that the former must possess more efficient DNA repair systems to counteract the enhanced mutation rates under the harsher environment. This review is focused on advances in understanding the function of some HA proteins in DNA repair and proposes directions for future studies of an endonuclease family.

Vogler et al. characterized a novel γ-carbonic-anhydrase (γ-CA) from a polyextreme Red Sea brine pool by single-cell genome sequencing. The enzyme was expressed in *Halobacterium* sp. NRC-1 and characterized by X-ray crystallography and mutagenesis. Several possible structural determinants responsible for the enzyme's salt stability were highlighted. The study reveals insights into the halophilic γ-CA activity and its unique adaptations, providing bases for outlining strategies for salt adaptation, underlying protein evolution mechanisms, and yielding proteins with industrially valuable properties.

The review by Verma and Satyanarayana discusses xylanolytic enzymes sources, characteristics, and applications obtained through metagenomic approaches, and their amelioration by genetic engineering. Xylanolytic enzymes working under extreme conditions, which are prevalent in bioprocessing industries, are in high demand; however, their availability does not match industrial requirements and the needs of the market. Since DNA manipulations and protein-engineering are quite unsatisfactory in generating extremophilic xylanolytic enzymes, metagenomic approaches have been successfully employed to uncover hidden genes that were inaccessible by culture-dependent approaches.

The study by Shahraki et al. identified and characterized cellulases by high-throughput metagenomic data, based on optimum temperature and pH using a sequence similarity-based annotation and an ensemble of supervised learning algorithms. Two enzymes from cattle rumen were cloned, expressed, and characterized. This study highlights the strength of machine learning techniques to predict enzymatic properties based on their sequence.

Brandt et al. screened a Vietnamese fungal culture collection and identified 12 highly active xylan degraders. One of the best producers was an *Aspergillus sydowii* strain. Illumina sequencing was used for strain identification and to determine differences with the CBS reference strain. With activity based on in-gel zymography and subsequent mass-spec identification, three potential proteins responsible for xylan degradation were identified and characterized. The active site residues in both enzymes were confirmed by site-directed mutagenesis. The results justify the classification of both xylanases as highly interesting for further development.

Dong et al. identified a putative gene encoding superoxide dismutase was identified in a thermoacidophilic *Alicyclobacillus* strain and characterized for its activity in the presence of metal ions and optimal conditions of pH and temperature, which implies striking potentials as a food additive and for medical use.

In James et al., a novel transketolase has been reconstituted from two polypeptide chains encoded by a “split-gene” identified in the genome of the hyperthermophilic bacterium *Carboxydothermus hydrogenoformans*. The reconstituted enzyme was biochemically characterized. This is the first example of a reconstituted “split-gene” transketolase to be characterized, allowing its evaluation for industrial biocatalysis.

Ding et al. cloned the CDF gene of *Thermoactinomyces vulgaris* encoding a subtilisin-like protease (Als) and expressed it in *Escherichia coli*. The recombinant enzyme was secreted as a mature form (mAls), was purified and characterized, and showed high compatibility with commercial laundry detergents. Due to its polyextremotolerant properties and keratinolytic capacity, Als may find applications in various industries such as laundry detergents, food processing, non-aqueous biocatalysis, and feather processing.

In conclusion, the search for new extremozymes, together with the study of their peculiar characteristics, is still a subject of great topicality involving numerous scientists investigating the microbial biochemical diversity and looking for new or improved applications. In any case, these studies widely contribute to a deeper understanding of the microbial world and its enormous potentialities.

## Author Contributions

MF and SG wrote the Editorial. All authors edited and commented on it.

## Conflict of Interest

The authors declare that the research was conducted in the absence of any commercial or financial relationships that could be construed as a potential conflict of interest.

## Publisher's Note

All claims expressed in this article are solely those of the authors and do not necessarily represent those of their affiliated organizations, or those of the publisher, the editors and the reviewers. Any product that may be evaluated in this article, or claim that may be made by its manufacturer, is not guaranteed or endorsed by the publisher.
